# Managing Food Allergies in Dining Establishments: Challenges and Innovative Solutions

**DOI:** 10.3390/nu17101737

**Published:** 2025-05-20

**Authors:** George N. Konstantinou, Ourania Pampoukidou, Daniel Sergelidis, Maria Fotoulaki

**Affiliations:** 1 Department of Allergy and Clinical Immunology, 424 General Military Training Hospital, 564 29 Thessaloniki, Greece; 2 Department of Food Safety Inspections of Animal-Origin and Composite Foods, Hellenic Food Authority (EFET), 570 01 Thessaloniki, Greece; parania2011@hotmail.gr; 3 Laboratory of Hygiene of Foods of Animal Origin-Veterinary Public Health, School of Veterinary Medicine, Aristotle University of Thessaloniki, 541 24 Thessaloniki, Greece; dsergkel@vet.auth.gr; 4 4th Department of Pediatrics, Papageorgiou General Hospital, School of Medicine, Aristotle University of Thessaloniki, 541 24 Thessaloniki, Greece; mfotoula@auth.gr

**Keywords:** food allergy, dining establishments, hidden allergens, cross-contamination, precautionary allergen labeling, VITAL scheme, staff training, real-time allergen detection, digital allergen management

## Abstract

**Background:** Food allergies represent a growing public health concern, with accidental exposures in dining establishments posing significant risks. Although various guidelines and interventions have been proposed, practical implementation remains challenging. **Objectives:** This narrative review aims to synthesize current evidence on major hazards in allergen management within dining settings and to evaluate emerging solutions designed to protect allergic consumers. **Methods:** A comprehensive literature search of peer-reviewed articles, surveillance reports, guidelines, and policy documents published in April 2025 was undertaken in PubMed, Scopus, and Web of Science. Studies were selected based on relevance to allergen management in dining establishments. An inductive thematic synthesis was performed, and a basic quality appraisal was conducted to prioritize stronger evidence. **Results:** Three major hazard themes—hidden allergens, cross-contamination during food preparation, and poor communication between staff and allergic consumers—were identified from the reviewed literature. Emerging interventions included enhanced staff training, improved allergen labeling practices, and the application of digital technologies such as smartphone apps and rapid allergen detection devices. However, inconsistencies in implementation and a lack of systematic validation limit the effectiveness of these approaches. **Conclusions:** Mitigating food allergy risk in dining establishments requires an integrated approach that combines strict kitchen controls with continuous staff education, transparent labeling, clear communication, and supportive policies. While promising interventions exist, more research is required to validate their effectiveness and to encourage standardized, widespread implementation to ensure the safety of individuals with food allergies.

## 1. Introduction

### 1.1. Food Allergies: A Growing Public Health Concern

The increasing prevalence of food allergies, particularly in developed countries, presents a significant challenge that lacks a definitive solution or cure, affecting millions of individuals with allergies and often leading to severe health consequences [1]. The World Health Organization (WHO) forecasts that nearly 50% of the global population will experience allergies by 2050, making allergies the fourth most common chronic disorder [2]. As a result, allergies represent a major public health concern that can impact individuals of all ages. Allergies are characterized as hypersensitivity reactions triggered by confirmed or strongly suspected immunological mechanisms against various allergens [3]. Allergens are antigens, substances, or environmental factors like house dust mites, pollen, or molds or proteins that can trigger allergic reactions [4].

According to the FDA, “…*food allergies occur when the body’s immune system reacts to specific proteins in foods*…” [5]. Different foods are considered allergenic sources, each containing one or more allergens. Food allergic reactions can vary in severity, ranging from mild symptoms like urticaria (hives) and angioedema (swelling) of the lips to severe, potentially life-threatening conditions known as anaphylaxis and anaphylactic shock, which may include fatal respiratory issues or cardiac arrest. Food allergy is part of a broader group of conditions called “food hypersensitivity”, including any adverse reactions to foods. If such adverse reactions are immune-mediated, they are classified as food allergies; if not, they are categorized as food intolerances, such as lactose or gluten intolerance [3].

### 1.2. Importance of Addressing Food Allergies in Dining Establishments

The overall economic impact of food allergies, including lost productivity and labor costs, is significant, estimated at around USD 24.8 billion annually in the United States [6]. Furthermore, the total number of anaphylactic reactions increased by 377% from 2007 to 2016 [7], with the United Kingdom experiencing an increase of 615% between 1992 and 2012 [8]. Additionally, food allergies negatively affect the quality of life of allergic individuals of all ages, both children and adults, as well as their families, requiring lifelong efforts to avoid the culprit allergens. This can lead to social exclusion, monotonous eating habits, food-related anxiety, and fear of trying new foods. Currently, about 33 million people in the U.S. suffer from food allergies, including 5.6 million children or about 1 in every 13 children [9,10].

Research indicates that about 74% of all food-allergic reactions involve non-prepackaged foods [11]. Furthermore, 59% of food-related anaphylactic hospitalizations in the United Kingdom occur in dining establishments [8]. Literature reviews emphasize the frequent presence of hidden allergens in commonly consumed foods, underscoring the need for allergic consumers to remain vigilant and cautious when dining out [12]. Given that most anaphylactic reactions take place outside the home, with only 7% happening at home [13], consumers must pay close attention and take appropriate precautionary measures (Table 1).

The confluence of these factors underscores a critical need to examine how the food service industry is adapting to protect individuals with food allergies. While numerous studies and guidelines have addressed specific aspects—from staff training to allergen labeling and new detection technologies—no comprehensive review has yet synthesized these insights across dining establishments. To address this gap, this narrative review integrates current evidence on the main operational challenges in managing food allergens (e.g., hidden ingredients, cross-contact, communication hurdles) and evaluates the effectiveness of emerging solutions. This approach highlights the best practices identified to date and the areas where knowledge remains limited, thus directly responding to the noted deficit in the literature.

## 2. Methods

### 2.1. Search Strategy

This narrative review adhered to the guidelines of the Scale for the Assessment of Narrative Review Articles (SANRA) [14]. Literature searches were conducted in PubMed, Scopus, and Web of Science using the following Boolean string:

(“food allerg*” OR “food hypersens*”) AND (restaurant* OR “food service” OR catering OR dining) AND (cross-contamination OR training OR labelling OR technology).

No language limits were set in this stage, but full texts had to be available in English for inclusion.

### 2.2. Eligibility Principles

A record was kept when it described allergen risk, management, or outcomes in any non-domestic eating setting (restaurants, cafés, catering, takeout, or institutional food service). We excluded single-sentence conference abstracts, duplicate datasets, and studies focused only on home cooking or primary food production. As this is a narrative review, we did not aim to provide an exhaustive list of every study; instead, we prioritized a breadth of perspectives while emphasizing timeliness and relevance to practical control measures.

### 2.3. Selection Process

Titles and abstracts were initially screened, followed by a brief full-text review. If two team members disagreed about including a paper, the senior author made the final decision. We maintained a final collection of publications that comprehensively covered epidemiology, training interventions, labeling policy, and emerging technology. A reference list of essential papers was carefully reviewed to ensure that no important guidance documents were missed.

### 2.4. Quality Appraisal

Narrative articles were evaluated according to the six items of SANRA [14] to identify weaknesses such as inadequate literature coverage or a lack of critical perspective. Empirical studies underwent a brief critical review based on three key questions: Was the method suitable for the question? Are the data clearly presented? Are the conclusions supported by the data? Sources that did not meet any of these criteria were still cited if they illustrated a historical point.

### 2.5. Synthesis

Key data (setting, hazard category, intervention, outcomes) were recorded in Excel and thematically synthesized into categories of hazards, interventions, and research gaps. Themes were identified inductively based on their frequent recurrence across multiple independent studies, reflecting their broad relevance and importance in managing food allergens in dining establishments. This inductive thematic approach allowed for the synthesis and clear representation of diverse and heterogeneous data from various contexts.

## 3. Challenges in Managing Food Allergies in Dining Establishments

The results highlight three primary hazards consistently emphasized across the reviewed studies: hidden allergens, cross-contamination, and communication barriers. Each theme reflects patterns frequently documented by multiple independent sources. Hidden allergens specifically emerged as the most commonly cited issue, reported by various studies due to undeclared ingredients or labeling inaccuracies [15,16,17,18,19,20,21,22,23,24,25,26,27,28,29,30,31,32]. Cross-contamination was another major concern thoroughly documented, stressing the risks associated with allergen transfer through shared equipment or poor practices [15,21,31,32,33,34,35,36,37,38]. While mentioned less often, communication barriers clearly surfaced as a significant hazard stemming from misunderstandings between allergic consumers and restaurant staff, as underscored by several focused investigations [39,40,41]. This thematic organization directly reflects the patterns and consensus emerging from the synthesized literature.

### 3.1. Hidden Allergens as a Major Challenge

Hidden allergens in dining establishments pose a significant risk to individuals with food allergies. These allergens are often concealed within dishes due to undeclared ingredients, cross-contamination during preparation, or misleading food labels. For example, certain sauces or dressings may contain nut or shellfish derivatives that are not evident from the menu description. Moreover, shared cooking equipment can introduce allergens into otherwise safe meals [15].

Dining establishments must implement comprehensive allergen management strategies to mitigate the dangers posed by hidden allergens. This includes thorough staff training to recognize and handle allergenic ingredients, a meticulous review of all recipes and food labels to ensure accurate allergen information, and clear communication with suppliers regarding ingredient contents. Regular audits of kitchen practices can help identify potential sources of cross-contamination, enabling establishments to take corrective actions promptly. By adopting these measures, restaurants can significantly reduce the risk of exposing customers to hidden allergens, thereby enhancing customer safety and trust [16].

Hidden allergens are a common cause of food allergies. They are called ‘hidden’ because detecting their presence in foods can be challenging, either because they are not recognized as allergens or because they might not appear on ingredient labels due to intentional or unintentional omissions (Table 2). Other factors include misleading labels, multiple names for the same ingredient, and cross-contamination during food preparation [17,18,19]. Additionally, ingredients intentionally added and labeled might be overlooked if they constitute a minor component of a complex dish, particularly given the variations in allergen labeling regulations across different countries [20].

Hidden allergens pose significant health risks for sensitized individuals, triggering reactions that range from mild symptoms like urticaria and angioedema to severe, potentially life-threatening reactions such as anaphylaxis [21]. Allergic reactions can occur even with small amounts of allergens, including through the inhalation of airborne food allergens or cooking fumes [22]. The exact prevalence of allergic reactions caused by hidden allergens is still unclear; however, a retrospective study indicated that hidden allergens accounted for 22.4% of food allergic reactions in individuals over 14 years old [23].

EU (EU) Regulation No 1169/2011 requires that consumers receive clear allergen information, including for non-prepacked foods, identifying the presence of any of the fourteen major allergens recognized by the European Food Safety Authority (EFSA) [24]. Despite this, incorrect or incomplete labeling continues, increasing risks for allergic consumers [25]. To mitigate these risks, the Rapid Alert System for Food and Feed (RASFF) has been established by the European Commission. RASFF quickly disseminates information on hidden allergens and food recalls, protecting public health across EU member states and associated countries such as Norway, Liechtenstein, Iceland, and Switzerland [26].

Effectively managing hidden allergens requires informed consumer behavior on multiple levels, starting locally with regular consultations of national authorities for guidance on food safety and allergen management practices (e.g., the Hellenic Food Authority known with the acronym EFET [27]). Nationally, consumers should work with allergists and local healthcare providers to keep their personal allergen information updated and receive personalized advice [28]. On an international level, staying informed through reliable platforms like the European Union’s RASFF provides critical updates on global product recalls involving undeclared allergens [18,29]. Additionally, vigilant practices such as carefully reading food labels and clearly communicating allergies when dining out remain fundamental for safely managing the risks of allergen exposure [30].

Allergen labeling varies by country, with common allergens like milk, eggs, gluten-containing cereals, crustaceans, peanuts, and tree nuts often recognized in most regulations. In contrast, allergens such as mustard, celery, mollusks, lupin, and buckwheat are limited to specific regions, reflecting varying dietary habits and exposure risks [31].

Notably, the frequency of undeclared allergens in products labeled as “allergen-free” reached 7.6%, highlighting significant risks for allergic consumers and underscoring the need for better nutritional education and increased awareness among manufacturers and regulatory bodies [18]. Global recalls from 2013 to 2019 identified milk as the most frequently undeclared allergen, often found in cereals, bakery products, and confectionery [32].

### 3.2. Cross-Contamination Risks

Cross-contamination poses a significant threat to individuals with food allergies in dining establishments. It occurs when allergenic foods inadvertently come into contact with allergen-free foods due to improper handling practices, inadequate cleaning, or shared utensils and cooking equipment. For instance, cutting boards, knives or spoons used for allergenic items like shellfish and peanuts can transfer allergens if subsequently used without thorough cleaning for foods intended to be allergen-free, such as vegetables or fruits [15]. A study conducted in the U.S. emphasized the significance of this issue, reporting that 53.9% of food allergy reactions occurring in restaurants happened despite customers providing prior notification to the restaurant staff, highlighting the persistent and widespread nature of cross-contamination [33].

Contamination risk arises throughout various stages of food preparation and service. Common problematic scenarios include salad bars, ice cream shops, and deli counters where serving utensils, scoops, and slicing equipment are frequently shared between allergenic and allergen-free products. Similar issues arise in manufacturing facilities, where production lines handle both allergenic items (like dairy-containing ice cream) and non-allergenic alternatives (such as dairy-free sorbet). Inadequate cleaning practices between production runs may result in allergen residues contaminating products that are supposedly allergen-free. Another significant cross-contamination source involves the use of shared frying oils, where allergenic foods like fish or crustaceans and allergen-free foods like french fries may be cooked consecutively, thereby increasing allergen exposure risk [21,32].

The uncertainty surrounding safe allergen threshold has led many food establishments to widely adopt precautionary allergen labeling (PAL), typically through labels such as “may contain traces” of allergens [34]. However, PAL implementation lacks regulatory standardization across countries. The absence of formal and consistent risk assessments prior to applying PAL frequently results in consumer confusion and inconsistent risk communication practices [31,35].

Effective cross-contamination risk assessment is challenging due to variability in allergen thresholds (Eliciting Doses: ED01, ED05, ED10), which vary considerably among individuals and can be affected by various factors, including the allergen itself, the nature of the food matrix, the amount consumed, and individual physiological conditions. Nonetheless, comprehensive and representative data on these thresholds remain limited, further complicating accurate cross-contamination risk assessments [34,35,36].

Initiatives such as the Voluntary Incidental Trace Allergen Labelling (VITAL) scheme by the Australian and New Zealand Allergen Bureau represent efforts to standardize PAL through formal allergen risk assessments [37]. Despite these efforts, a universally harmonized standard remains elusive. International regulatory bodies, notably the Codex Committee on Food Labelling, a joint FAO/WHO committee, are actively reviewing the evidence to develop globally applicable guidelines for PAL addressing existing inconsistencies among various jurisdictions, including those within the European Union [31,34].

Building on these regional efforts, the Codex Committee on Food Labelling and the 2023 FAO/WHO Expert Consultation on Risk Assessment of Food Allergens (Part 3) have suggested a tiered, reference-dose method for PAL. The FAO/WHO report advises (i) establishing quantitative action levels based on the ED05 or a suitable lower reference dose for each priority allergen, (ii) making PAL mandatory when the estimated unintentional presence of allergens surpasses that action level and prohibiting its use when levels are below it, and (iii) adopting a single, standardized phrase, “May contain [allergen],” to replace the current array of voluntary statements. The consultation also calls on Codex to incorporate these recommendations into an updated General Standard for the Labelling of Pre-packaged Foods, thus providing a globally harmonized and enforceable framework for future PAL implementation [38].

Food-borne anaphylaxis typically results from the accidental ingestion of allergenic foods that exceed an individual’s tolerance threshold. While the labeling of the 14 major allergens as ingredients is mandatory in the EU, there is no legal obligation to clearly label potential cross-contaminants. As a result, precautionary statements like “may contain traces” are often insufficient for allergic individuals as they do not indicate specific contamination levels in relation to personal allergen thresholds [34,39].

To effectively reduce cross-contamination risks, dining establishments must implement strict allergen management protocols. This includes dedicating separate preparation areas and utensils exclusively for allergen-free meal preparations, thoroughly cleaning cooking equipment between uses, and continuous staff training focused on recognizing and proactively preventing potential cross-contact scenarios. Regular audits and strict adherence to established food safety standards further reinforce these measures, significantly reducing the risk of accidental allergen exposure and enhancing customer safety and confidence [16].

### 3.3. Communication Barriers

Effective communication between restaurant staff and customers with food allergies is crucial for ensuring safe dining experiences. However, barriers such as inconsistent or unclear communication protocols, insufficient staff awareness, inadequate allergen-specific training, and cultural or language differences between customers and staff frequently result in misunderstandings and allergen exposure risks. Such communication breakdowns have consistently been identified as a major cause of food allergy reactions in restaurants [40,41,42].

To overcome these barriers, restaurants should establish standardized communication protocols, conduct regular allergen awareness training, encourage open dialog between customers and staff, and utilize visual aids such as allergen charts or clearly marked menus [42].

### 3.4. General Recommendations for Safe Dining Practices

Ensuring a safe dining experience for individuals with food allergies involves careful consumer practices combined with diligent restaurant procedures. Customers should clearly communicate their allergies to restaurant staff, preferably by using tools like a “chef’s card” or digital applications specifically designed to convey allergen information [42]. It is crucial for consumers to inquire about ingredients and preparation methods to prevent cross-contamination. Foods with a higher risk of cross-contact, such as fried items, should be avoided unless safety is confirmed. Desserts often pose a risk due to hidden allergens; therefore, caution is advised, and selecting safe desserts at home may be a better option. Additionally, consumers should regularly consult reliable resources and allergist recommendations to stay informed about potential allergen risks [28].

Dining establishments should adopt structured procedures for managing allergens. These include clearly labeling allergen-free options, thoroughly training all staff to handle allergen inquiries effectively, and maintaining rigorous kitchen hygiene standards to prevent cross-contact. Clear and visible allergen charts and menus improve communication and assist both customers and staff in making informed decisions, thus significantly reducing accidental allergen exposure and enhancing the dining experience [43].

Customers should always read food labels carefully to verify the absence of allergens before consumption. Utensils, cutting boards, and cooking pans must be cleaned thoroughly with soap and water before and after use. Establishments should use separate utensils and dishes for allergy-safe foods, ideally employing different colors to easily distinguish safe kitchen tools. It is recommended that allergy-safe foods be prepared first when multiple dishes are being cooked. Foods intended for allergic consumers should remain covered and separate from other dishes to prevent contamination [16,44].

If there is accidental contamination, the entire dish must be discarded and remade, as even trace amounts of an allergen can make a meal unsafe. Individuals handling allergenic foods must wash their hands with soap and water before touching anything else; hand sanitizers or plain water are insufficient for allergen removal. Surfaces like counters and tables should be cleaned with soap and water or disinfectant cleaners using disposable towels. All utensils, pots, and pans should be washed in a dishwasher or cleaned with hot water and soap and then air-dried. Sharing food, drinks, or utensils is discouraged, especially for children, who should be educated accordingly to prevent allergen exposure at school or during social interactions [16,44,45,46].

## 4. Innovative Solutions

**Figure 1:**
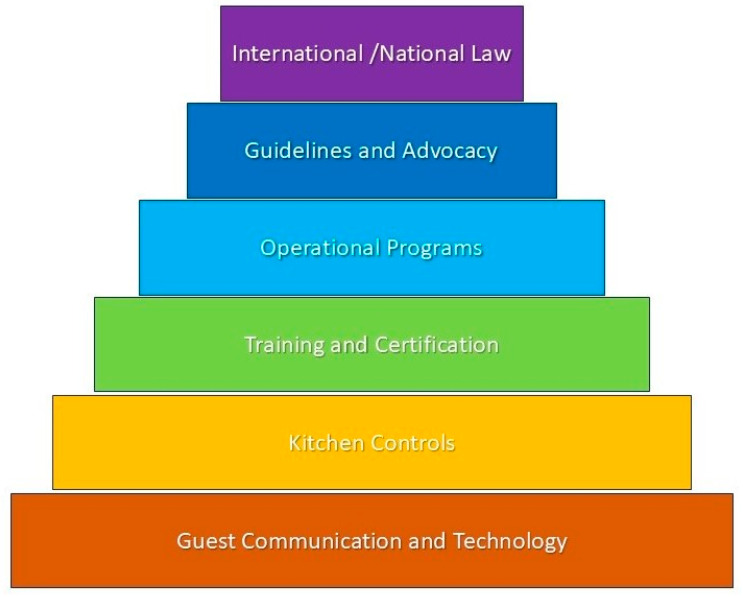
Six-tier hierarchical framework for managing food-allergy risk in dining establishments, showing how international regulation cascades through advocacy, operational programs, staff training, kitchen controls, and guest-facing communication. *International/National Law*—binding legal requirements (e.g., EU 1169/2011). *Guidelines and Advocacy*—voluntary standards and lobbying that shape best practice (GA^2^LEN, FARE). *Operational Programs*—site-level management systems, SOPs, and digital allergen dashboards. *Training and Certification*—formal staff courses such as FARECheck or ServSafe Allergens. *Kitchen Controls*—engineering and administrative barriers (separate prep areas, validated cleaning). *Guest Communication and Technology*—clear menu icons, PAL wording, and QR/app tools that inform diners.

Before detailing individual interventions, it is useful to frame them within the broader six-tier hierarchy of allergen-risk governance illustrated in Figure 1. This novel framework traces the cascade from binding international and national legislation (Tier 1), through expert guidelines and advocacy networks (Tier 2) and site-level management programs (Tier 3), to staff certification (Tier 4), kitchen controls (Tier 5), and guest-facing communication technologies (Tier 6). By visualizing how top–down policy and organizational support enable frontline practice, the figure clarifies where the solutions discussed in Section 4.1, Section 4.2, Section 4.3 and Section 4.4 fit and where innovation is still needed to close residual safety gaps.

### 4.1. Staff Training and Education Programs

Comprehensive training and education programs for restaurant staff are essential for effectively managing food allergies and ensuring customer safety. Staff should possess extensive knowledge of common food allergens, including milk, eggs, fish, shellfish, tree nuts, peanuts, wheat, and soy, and less common allergens that may pose risks to specific populations or regions. This understanding enables staff to accurately identify potential allergens in dishes and ingredients, improving their ability to confidently address consumer concerns [47,48].

Training programs must emphasize procedures to prevent cross-contact between allergenic and non-allergenic foods. This involves utilizing designated preparation areas, utensils, and equipment specifically reserved for allergen-free meals. Effective communication training is also crucial; staff should actively engage customers regarding dietary restrictions, clearly communicate ingredient information, and ensure consistent and accurate communication between kitchen staff and servers to avoid misunderstandings [15].

Another critical aspect of staff training is emergency preparedness. Staff members should be trained to quickly recognize signs of allergic reactions and to implement appropriate emergency responses, such as administering epinephrine and contacting emergency services when necessary [49]. Regular drills and refresher courses can reinforce this training, ensuring a high level of staff readiness [30,50].

Several structured and specialized training programs, including the “FARECheck Instructor Training” offered by FARE [51] and the “ServSafe Allergens” course provided by the National Restaurant Association [52], support dining establishments in implementing consistent allergen management practices. These programs focus on practical strategies for allergen recognition, effective response to allergic reactions, accurate ingredient identification, minimizing cross-contact risks, and clear communication about allergen risks. Regular updates and assessments through these training initiatives keep restaurant staff informed about emerging best practices and allergen management strategies, thereby significantly enhancing dining safety and customer satisfaction.

### 4.2. Allergen-Free Menu Development

The development of allergen-free menus is a crucial step toward safely accommodating diners with food allergies. Restaurants must clearly identify prevalent allergens and develop alternative recipes that either exclude these allergens or substitute them with safe, non-allergenic ingredients. For example, almond or oat milk can effectively replace cow’s milk, and chickpea or rice-based pasta can substitute wheat products [53,54]. Ensuring these ingredients are genuinely allergen-free requires close collaboration with suppliers and regular quality audits to prevent accidental cross-contamination during production, processing, or delivery [48].

Clear, accurate, and detailed menu labeling is essential. Menus should explicitly highlight allergen-free items using standardized symbols or distinct formatting to enhance clarity and ease of identification by consumers [24,43]. Providing comprehensive ingredient lists and transparent preparation methods allows customers to make informed and safe dining decisions [18]. Additionally, staff training in understanding and communicating allergen-free menu options is vital, ensuring that restaurant personnel confidently advise customers and manage allergen-free orders appropriately [40,50]. These measures collectively strengthen consumer trust, enhance the restaurant’s reputation for inclusivity, and significantly reduce the risk of allergic reactions.

### 4.3. Technological Interventions

Incorporating technological solutions into restaurant operations greatly enhances allergen management, boosts safety, and improves communication. Digital allergen management systems like TrustDish allow restaurants to keep precise, real-time allergen data available to both staff and customers [55]. Sophisticated artificial intelligence (AI) systems enable automated detection and prediction of allergens in ingredients and finished meals, minimizing human error and improving safety [56].

Modern Point-of-Sale (POS) systems with integrated allergen alert features enable the immediate identification of allergenic risks and offer real-time updates about menu changes. Additionally, customer-facing mobile applications, like AllergyEats [57], Spokin [58] and similar platforms, empower diners by providing real-time allergen information, restaurant reviews, and recommendations for safe dining. Such technologies significantly support informed decision-making for customers with food allergies (Table 3).

Portable allergen detection devices are under active investigation as tools to enhance food safety in real-world settings. The original Integrated Exogenous Antigen Test (iEAT), developed at Harvard Medical School, introduced a portable electrochemical platform capable of detecting common food allergens such as peanuts and gluten within minutes using magnetic beads and smartphone integration [59]. Building on this, the next-generation iEAT2 added a built-in grinding mechanism and a 16-electrode array for multiplex detection, showing high sensitivity for several allergens in under 15 min [60]. Both systems remain research prototypes and are not FDA-approved or commercially available. A different technological approach involves the use of molecularly imprinted polymers (MIPs) for electrochemical allergen detection [61]. A MIP-based sensor developed as part of the Allergy Amulet platform was tested across over 40 complex food samples, demonstrating accurate detection of soy allergens even in processed foods, with better performance than commercial lateral flow devices (LFDs) in certain contexts [62]. Meanwhile, the Nima Sensor, a consumer device using a single-use capsule with a lateral flow strip and optical detection, has shown reliable gluten detection at ≥20 ppm but limited sensitivity in some complex matrices [63]. Overall, while these devices highlight promising directions for on-site allergen detection, their current utility in dining establishments is constrained by technological limitations, cost, regulatory status, and the need for further validation in real-world environments.

### 4.4. Policy Implementation and Advocacy

Robust allergen management policies and active advocacy are vital elements of a comprehensive strategy to improve food allergy safety in dining establishments. Clear, documented policies that outline protocols for allergen identification, labeling, cross-contact prevention, staff responsibilities, and emergency response procedures provide consistency in allergen management. These policies should be reviewed and updated regularly to align with evolving scientific evidence and regulatory requirements [64].

Advocacy efforts by organizations like FARE are essential for shaping legislative initiatives, establishing industry-wide best practices, and raising public awareness regarding food allergies [65]. Advocacy groups actively work with policymakers, regulatory bodies, industry representatives, and consumer groups to promote safer dining environments through improved legislation and increased public education campaigns [66,67,68,69,70,71].

Restaurants and food service establishments should actively participate in these advocacy initiatives, supporting the development of clear and consistent allergen management standards across the industry. By adopting rigorous allergen management policies and contributing to advocacy efforts, dining establishments demonstrate a strong commitment to customer safety, promote greater awareness of food allergies, and enhance their reputation among customers and within the broader community [24,43,71,72,73,74,75].

## 5. Case Studies and Real-World Applications

### 5.1. Success Stories in Allergen Management

Implementing comprehensive allergen management strategies in dining establishments has led to significant improvements in customer safety and satisfaction. For instance, integrating food management safety into computerized foodservice systems, highlighting labels on food trays, introducing safety checks in food delivery processes, and ensuring the nutritional requirements of patients with allergy restrictions have proven effective. These measures, incorporated into written protocols and supported by staff training, have promoted food allergy safety in hospital settings [76].

### 5.2. Incidents and Lessons Learned

Despite increased awareness, incidents of severe allergic reactions in dining establishments continue to occur, underscoring the need for stringent allergen management practices. A cross-sectional analysis conducted in the Qassim region of Saudi Arabia assessed the knowledge, practices, and attitudes of restaurant staff regarding food allergies. The study revealed that most restaurant staff had limited knowledge of food allergens and their symptoms, with only a small proportion having received specific training on food allergies. Moreover, only 14% of restaurants provided allergen information on their menus. Despite these knowledge gaps, most staff exhibited positive attitudes toward managing food allergies. These findings highlight the urgent need for the restaurant sector to implement food allergy measures, including clear policies and comprehensive training, to prevent potentially life-threatening incidents [77].

### 5.3. Technological Innovations in Allergen Management

The rise in mobile applications provides promising tools for self-managing food allergies. A systematic search and quality assessment of these apps revealed that while the overall quality is acceptable, improvements are needed in user engagement, especially for apps focused on food products and restaurant information. Enhancing the functionality and reliability of these applications could help individuals make informed dietary choices and manage their allergies more effectively [78].

### 5.4. Policy Development and Ethical Considerations

Developing comprehensive food allergy policies ensures a consistent approach to managing allergens in dining establishments. Compliance with ethical principles like confidentiality, fairness, and empowerment is vital in policy development. Structuring policies around these ethical principles offers guidance for school health officials and can be adapted for use in dining establishments to support better policy-making and implementation [79].

### 5.5. Regulatory, Supply Chain, Technological and Economic Barriers

While the prior case studies showcase successful initiatives alongside persistent challenges in managing allergens, their overall impact is significantly shaped by systemic barriers. This section shifts focus from successful strategies to the factors that hinder widespread adoption and scalability. We analyze four primary domains of constraint—regulatory fragmentation, supply chain vulnerabilities, technological/approval hurdles, and economic pressures—alongside a fifth, the cross-cutting consumer-centered perspective, which influences all stages of allergen risk management. The subsections that follow unpack the four domains in detail, beginning with the complexities of international labeling and travel-related risks. Table 4 summarizes each domain (plus the consumer-centered layer), pairing representative interventions with the key challenges that continue to hinder their effective implementation.

#### 5.5.1. Regulatory Fragmentation and International Travel

Despite the increasing availability of portable allergen detection devices and digital allergen management platforms, real-world adoption in dining establishments remains limited. A significant barrier is the absence of harmonized international standards for allergen labeling, threshold levels, and priority-allergen lists [15,20]. This regulatory fragmentation results in a patchwork of requirements across jurisdictions, complicating the development and global deployment of these technologies. This problem is magnified when food-allergic consumers travel. They must interpret anything from voluntary, non-quantitative “may contain” statements common in the EU and North America to Japan’s legally enforced 10 mg/kg PAL threshold for several allergens. Dinardo et al. [80] show that such variability confuses consumers and may lead to either unnecessary dietary restrictions or unrecognized exposure, while Chang et al. [20] highlight the added burden of foreign languages, unfamiliar icons, and inconsistent PAL prominence.

Japan operationalizes such thresholds with a nationally standardized toolkit of ELISA, Western blot, and PCR protocols, published by the Ministry of Health, Labour and Welfare and updated annually to reflect new matrices and detection limits [81,82]. These validated methods can quantify allergen traces at or below 10 µg/g, providing the analytical certainty needed for enforceable PAL, and highlighting the Japanese model as proof of concept that micro-quantity thresholds, coupled with harmonized laboratory guidance, can improve both industry compliance and consumer confidence.

Until a globally aligned reference-dose framework emerges, pre-travel counseling should include country-specific labeling rules, the carrying of dual epinephrine autoinjectors, and the selection of allergen-aware airlines and accommodations.

#### 5.5.2. Supply Chain Shocks and Botanical Impurities

Recent geopolitical conflicts, pandemic-related logistical bottlenecks, and crop failures caused by climate change have exposed a new risk of unintentional exposure: botanical impurities. These are unintended plant-derived contaminants that infiltrate raw materials at earlier stages. Additionally, the conflict between Russia and Ukraine has led to a transition from sunflower oil to alternative sources, which has introduced new allergen risks within supply chains in areas prone to peanut or soy cross-contamination, like India. Dinardo et al. describe multiple such incidents and conclude that botanical impurities now constitute a significant emergent allergenic hazard in global supply chains [83]. Because food service operators cannot control these upstream substitutions, robust supplier-verification programs and contingency labeling protocols are essential components of any allergen-control plan, particularly for businesses serving traveling consumers with food allergies.

#### 5.5.3. Technological and Regulatory-Approval Hurdles

Regulatory inconsistency also affects device approval. The Nima handheld sensor, for example, could be sold in the United States without pre-market FDA review, while a comparable product in the European Union would require CE marking under the Medical Device Regulation. Generating the analytical performance and clinical relevance data required for such approval is costly and time-consuming, which discourages small innovators.

The same oversight gap applies to smartphone-based digital allergen managers: only a small fraction are registered as medical devices, and independent audits reveal false-negative rates of up to 20% for traces of peanuts, milk, or gluten—statistics that might create a false sense of security for users [84]. Moreover, most apps are offered only in English, rely on stable internet for label decoding, and require a level of digital literacy that could alienate older adults or travelers in areas with low bandwidth. These technical and accessibility challenges further diminish the real-world effectiveness of otherwise promising tools [85].

#### 5.5.4. Economic Constraints

Economic constraints exacerbate these barriers. Handheld readers cost over USD 400, with single-use cartridges priced up to USD 10, and subscription platforms for digital allergen menus involve ongoing fees and staff time. For many venues operating on tight margins, such investments are not financially feasible without external incentives or regulatory mandates. As a result, advanced allergen risk management tools remain disproportionately concentrated in larger chain restaurants or institutional settings, where economies of scale and centralized policies can support their implementation.

## 6. Future Directions and Recommendations

### 6.1. Research Gaps

Despite significant progress in allergen management, several research gaps remain. One critical area requiring further investigation involves the effectiveness of staff training programs in real-world scenarios [86]. Comprehensive evaluations are needed to determine how different training methodologies impact staff behavior, customer safety, and the frequency of allergic reactions in dining establishments. Furthermore, standardized metrics for evaluating training outcomes should be established to allow for consistent assessment across different contexts studies [50,87].

Another research gap concerns the development and validation of universally applicable communication protocols. There is limited evidence assessing the effectiveness of current communication strategies in reducing allergen exposure incidents, especially across various cultural and linguistic contexts. Studies should focus on identifying best practices for clear and efficient allergen communication, ensuring that customers fully understand the risks associated with dining choices [48].

Technological interventions in allergen management represent another significant area that currently lacks thorough evaluation. Future studies need to systematically evaluate the accuracy, reliability, and usability of digital allergen management systems and mobile apps. It is crucial to explore user acceptance, cost-effectiveness, and the overall influence of these technologies on allergen safety to promote wider adoption and enhance public health outcomes [88,89,90].

Additionally, robust research into methods of cross-contamination prevention and accurate allergen detection is needed. This includes studies assessing the effectiveness of existing cleaning protocols, specialized allergen-free equipment, and cutting-edge detection methods such as portable allergen sensors. Understanding the practical limitations and potential improvements of these methods can significantly enhance allergen safety in dining establishments [91].

The described evidence primarily focuses on North American and European practices. A supplementary search targeting Asia, Latin America, and Africa retrieved only a handful of small, descriptive studies (e.g., staff-knowledge surveys from Malaysia, Bangladesh, Kuwait, Saudi Arabia, and Brazil) with limited methodological rigor and heterogeneous outcome measures [77,92,93,94,95,96,97]. Because these data do not yet allow for robust, region-specific best-practice recommendations, the present review necessarily relies more heavily on the mature literature from North America and Europe while highlighting the urgent need for well-designed studies in under-represented settings.

### 6.2. Recommendations for Stakeholders

Restaurant managers and food service providers play a central role in allergen safety and should prioritize the continuous training of staff regarding allergen awareness, management strategies, and emergency response [98]. They must ensure transparency by clearly and accurately labeling all menu items and promptly communicating ingredient changes effectively [99]. Restaurants should implement standardized protocols for managing allergens and perform regular audits to identify and address potential safety gaps proactively.

Policymakers and regulatory bodies should strengthen and enforce regulations concerning allergen labeling and management practices. Clear guidelines and mandatory allergen management training for restaurant staff can significantly reduce risks [100].

Public education campaigns led by regulatory authorities and advocacy groups can also raise awareness among consumers and the broader community about food allergies, fostering a more supportive and informed dining experience culture [101].

Consumers should take an active role in their allergen safety by clearly communicating their dietary restrictions when dining out [102]. Using available resources, such as allergen management apps, chef cards, and official health advisories, can empower customers to make informed and safe dining decisions. Continued advocacy from consumers can drive further improvements and policy changes, enhancing the overall safety of the dining experience [103].

### 6.3. Recommendations for Future Research

Future research should focus on evaluating the long-term effectiveness and sustainability of staff training programs in reducing allergic incidents. Comparative studies that assess different training methodologies and their outcomes will offer valuable insights into optimizing allergen management training [104].

Further research into standardized communication protocols that address cultural and language differences is essential. Developing clear and universally applicable guidelines will improve allergen information transfer, reducing risks associated with miscommunication [105].

Investigating technological solutions, such as digital platforms and real-time allergen detection devices, is essential for understanding their practical benefits and limitations. Research must prioritize the validation of these technologies, examine their cost-effectiveness, and evaluate their effects on operational efficiency and consumer trust [106].

Finally, research on preventing cross-contamination should continue to refine current cleaning and preparation methods while exploring innovative approaches. Developing clear threshold levels for allergen exposure and reliable detection methods will further enhance allergen safety protocols. Addressing these research gaps will significantly contribute to safer dining experiences for individuals with food allergies [44,107,108].

It is also important to recognize that not all studies correspond with the positive outcomes highlighted in our review. For example, even with formal allergen policies in place, allergic reactions in restaurant settings remain disturbingly frequent [8,11,12,13]. Similarly, precautionary allergen labeling (e.g., “may contain” warnings) is widespread, but its implementation is often inconsistent and does not always lead to a measurable decrease in adverse events [34,39]. These mixed findings highlight the complexity of managing food allergies and warn against overgeneralizing successes. By openly acknowledging such differing results and limitations, we offer a more balanced perspective and reduce potential bias, emphasizing that while many interventions show promise, their real-world impact can differ and requires further study. Given these complexities, it is important to consider the nature and limitations of this review.

The narrative nature of this review indicates that, unlike a systematic review, it does not claim to be exhaustive. However, the transparent search terms, defined inclusion criteria, and clear two-step quality screening provide the reader with a solid understanding of how evidence was selected and prioritized. Papers addressing the three fundamental questions (see the last paragraph of Section 2.4) were given greater emphasis in drawing conclusions, while those with methodological gaps were mainly cited for context. This graded use of the literature, along with clear acknowledgment of contradictory findings, helps reduce the risk of bias that can occur when a narrative review relies too much on any single strand of evidence.

## 7. Conclusions

Effectively managing food allergies in dining establishments requires an integrated, multi-stakeholder approach that encompasses rigorous kitchen protocols, continuous staff training, transparent communication, and supportive public policy. Hidden allergens, cross-contamination, and communication lapses continue to be the primary causes of accidental reactions—yet case studies indicate that targeted interventions, such as structured training programs like ServSafe Allergens and FARECheck, along with digital allergen-tracking platforms and emerging point-of-service detection devices, can significantly reduce risk while maintaining operational efficiency. Looking forward, harmonized global standards for precautionary allergen labeling, clearer threshold guidance, and validated real-time sensing technologies will be crucial in closing existing safety gaps. Simultaneously, longitudinal research connecting specific training and communication strategies to measurable reductions in adverse events will enhance the evidence base for best practices. By embracing innovation, enforcing robust policies, and equipping both food service professionals and consumers with reliable information, the restaurant industry can transform allergen management from a persistent challenge into a model of proactive public health stewardship, ensuring that every guest, regardless of dietary restriction, can dine with confidence and dignity.

## Figures and Tables

**Table 1:** Food allergy burden at a glance.

Indicator	Figure	Reference(s)
Global share of population projected to have any allergy by 2050	≈50%	[2]
Annual U.S. economic impact of food allergy	USD 24.8 billion	[6]
Increase in anaphylactic reactions (U.S., 2007 → 2016)	+377%	[7]
Increase in anaphylaxis-related hospitalizations (UK, 1992 → 2012)	+615%	[8]
People in U.S. living with food allergy	33 million (5.6 million children)	[9,10]
Reactions linked to non-pre-packaged foods	74%	[11]
UK food-related anaphylactic hospitalizations that occur in dining venues	59%	[8]
Share of reactions that happen at home	7%	[13]

**Table 2:** Hidden allergen “hotspots” in commercial kitchens.

Allergen	Typical Dish/Component Where it Hides	Why It Is Missed	Simple Mitigation
Milk	Brioche buns, mashed potatoes (butter), “non-dairy” chocolate	Name is not explicit; carry-over via dairy fats	Dairy-only prep area; segregated spatulas
Peanut	Satay sauce in dressings, chili pastes, desserts with “groundnuts”	Alternate naming; imported ingredients	Supplier audit; staff cue-cards on aliases
Fish	Caesar dressing (anchovy), Worcestershire sauce, frying oil shared with fish	Compound ingredients; shared equipment	Dedicated fryers; clean-in-place verification
Gluten	Soy sauce in marinades, meat substitutes	Composite ingredients not declared	Switch to GF-certified tamari; QC swabs

GF = gluten-free (i.e., the tamari has been independently certified to contain no detectable gluten). QC = quality control (swabs used after cleaning to verify that allergenic proteins or general soil have been removed).

**Table 3:** Portable and digital allergen detection technologies (April 2025 landscape).

Platform/Device	Targets	Time to Result	Detection Limit	Readout/Interface	Stage (Apr 2025)	Key limitation Noted in Trials
iEAT (Harvard prototype)	Peanut, gluten	≈10 min	<5 ppm	Smartphone app; electro-chemical	Lab prototype	Requires sample dilution and external magnet
iEAT 2	8 top allergens	<15 min	<1 ppm for peanut	On-board grinder; 16-sensor array	Research prototype	Not FDA-cleared; high unit cost
Allergy Amulet (MIP sensor)	Soy (pilot), peanut (R&D)	2–3 min	0.7 ppm soy	Wearable key fob + phone app	Beta trials	Needs single-use test strip; matrix interference in oily foods
Nima Sensor	Gluten; peanut module shelved	2–3 min	≥20 ppm gluten	LED + Bluetooth to app	Limited retail	False negatives in hydrolyzed proteins
TrustDish (SaaS)	All EU-14 + sesame	Real-time	Database driven	Cloud dashboard and QR menu	Commercial EU/UK	Relies on staff upkeep of ingredient data

SaaS = Software as a Service: a cloud-computing model in which the software application is hosted by a provider and accessed by customers over the internet, typically through a web browser and paid for by subscription rather than installed locally.

**Table 4:** Key domains limiting widespread allergen risk management and their associated interventions and challenges.

Intervention Domain	Examples of Specific Measures	Principal Challenges
Regulatory harmonization	Codex/FAO-WHO threshold-based PAL National reference doses Standardized analytical protocols (e.g., in Japan)	Global patchwork of PAL wording and action levels Slow legislative uptake
Supply chain controls	Robust supplier-verification programs Contingency labeling for substitutions Upstream testing for botanical impurities	Geopolitical shocks Hidden botanical impurities
Technological solutions	Portable sensors Smartphone allergen-scanner apps Cloud-based menu databases	False negatives; uneven regulatory oversight Language, connectivity, and digital literacy gaps
Economic/ organizational levers	Chain-wide policies and training Subsidies or tax incentives for small venues	High device and cartridge costs Staff turnover; training burden
Consumer-centered strategies	Pre-travel counseling Translation/allergen cards Dual epinephrine carriage	Information overload abroad Variable label formats and icons

## Data Availability

No new data were created or analyzed in this study. Data sharing is not applicable to this article.

## Abbreviations

AI	artificial intelligence
Codex	Codex Alimentarius Commission
EFSA	European Food Safety Authority
FDA	U.S. Food and Drug Administration
FARE	Food Allergy Research and Education
PAL	precautionary allergen labeling
RASFF	Rapid Alert System for Food and Feed
VITAL	Voluntary Incidental Trace Allergen Labelling
WHO	World Health Organization
